# Methylliberine Ingestion Improves Various Indices of Affect but Not Cognitive Function in Healthy Men and Women

**DOI:** 10.3390/nu15214509

**Published:** 2023-10-24

**Authors:** Michael B. La Monica, Betsy Raub, Keeley Malone, Shelley Hartshorn, Jodi Grdic, Ashley Gustat, Jennifer Sandrock

**Affiliations:** The Center for Applied Health Sciences, Canfield, OH 44406, USAsh@thecahs.com (S.H.); ag@thecahs.com (A.G.);

**Keywords:** cognitive flexibility, mood, energy, concentration, motivation, nootropics

## Abstract

This study assessed the acute effects of oral methylliberine (Dynamine^TM^) supplementation on cognitive function and indices of well-being. This was a double-blind, randomized, within-subject crossover trial. In total, 25 healthy men and women (33.5 ± 10.7 yr, 172.7 ± 8.6 cm, 73.3 ± 11.0 kg) underwent pretesting before ingesting methylliberine (100 mg) or a placebo (PLA) for 3 days. On the fourth day, the participants were tested before their fourth dose (baseline) and every hour post-ingestion for 3 h. After a one-week washout period, the participants repeated testing with the alternate investigational product. The testing battery consisted of vitals, Stroop test, Trail Making Test-B, and visual analog scales that assessed various indices of well-being. Mixed factorial ANOVAs with repeated measures were used to assess all variables. There were significant (*p* ≤ 0.050) interactions in terms of concentration, motivation, and mood. Methylliberine improved concentration at 1 and 3 h, motivation at 3 h, and mood at 1, 2, and 3 h (*p* ≤ 0.050). Methylliberine improved energy, sustained energy, and mood in all participants to a greater extent than PLA at 1 h and 3 h relative to baseline (*p* ≤ 0.050). PLA improved motivation at 1 and 2 h and mood at 2 h (*p* ≤ 0.050). Methylliberine improved concentration, well-being, and the ability to tolerate stress to a greater extent than PLA at 3 h relative to baseline (*p* ≤ 0.050). Women observed elevations in sustained energy at 1 and 3 h (*p* ≤ 0.050) with methylliberine vs. PLA. Methylliberine had a negligible influence on cognitive function and vitals (*p* > 0.050), and no adverse events were reported. Methylliberine significantly improved subjective feelings of energy, concentration, motivation, and mood, but not cognitive function. PLA improved motivation and mood at hours 1 and 2, while methylliberine sustained these benefits for longer. Methylliberine also improved concentration, well-being, and the ability to tolerate stress to a greater degree than PLA, while having no detrimental effects on vital signs. Methylliberine also seemed to have a positive impact on sustained energy in women.

## 1. Introduction

Natural nootropics are organic substances that may act as a vasodilator in the brain, increase cellular energy, and/or protect the brain from oxidative stress [1,2]. Nootropics may also improve cognitive function, reduce tiredness, improve reaction time, improve memory retention and recall, and reduce mental fatigue [1,2]. The most widely consumed nootropic is caffeine, and there is an abundance of research demonstrating improved attention and executive function; however, in some individuals, caffeine use can have undesirable affects such as a crash, jitteriness, anxiety, or elevated vital signs [3,4]. Therefore, natural substances that can provide similar cognitive enhancements without the side effect profile of caffeine may be desirable for consumers. Methylliberine (trademark name Dynamine^TM^) is a purine alkaloid metabolite of caffeine which may provide similar cognitive enhancements as caffeine with none of the hemodynamic effects (i.e., elevated blood pressure and jitteriness) [5]. A few studies have examined the impact of the combination of caffeine, theacrine (as TeaCrine^®^), and methylliberine (CMT) [5,6,7] on cognitive tasks, but investigations examining methylliberine independently are scarce. To date, only two investigations have analyzed independent methylliberine ingestion in humans. These investigations conducted safety profiles (i.e., cardiovascular function and comprehensive hematological panel) of methylliberine supplementation alone and in conjunction with TeaCrine^®^ and/or caffeine over an acute period of 48 h and over a period of four weeks in healthy young men and women [8,9]. Preceding human trials, methylliberine was shown to be safe in rats following chronic dosing (90 days) and after a 28 day follow-up [10].

Several studies have examined the impact CMT has on cognitive performance. A previous study from La Monica et al. (2021) comparing a placebo (PLA), caffeine, and CMT on gaming performance in a first-person shooter with recreational gamers showed that CMT, not CAFF, improved the time it took to eliminate a target vs. PLA. Simultaneously, caffeine increased jitteriness relative to baseline and was not able to maintain cognitive control vs. CMT and PLA [6]. It was also noted that CMT and CAFF elevated systolic blood pressure (SBP) slightly vs. PLA relative to baseline [6]. Notably, the subjects perceived themselves to perform better during the gaming simulation under the CMT treatment vs. PLA relative to baseline [6]. Likewise, Tartar et al. (2021) compared CMT, caffeine, and PLA in amateur gamers and found that CMT and PLA improved inhibitory control on a Flanker test vs. caffeine. CMT also improved subjective alertness vs. PLA and improved reaction time on the Psychomotor Vigilance Task relative to baseline, whereas caffeine and PLA did not [7]. In addition, CMT (relative to caffeine) was associated with lower self-reported headaches [7]. Unique to Tartar et al. (2021), the investigation observed an increase in delta power during EEG recordings under the CMT treatment, while caffeine showed a decrease in delta power relative to baseline [7]. The authors concluded that an increase in delta power along with a possible increase in theta power (in CMT) were associated with an increase in attention and cognitive control [7,11,12]. Lastly, Cintineo et al. (2022) compared CMT, caffeine, and PLA on reaction time and marksmanship in tactical athletes and found that caffeine and CMT (as opposed to PLA) were able to improve reaction time in a vigilance task; however, there were no differences in accuracy or reaction time between treatments within a marksmanship task. Also, caffeine elevated systolic and diastolic blood pressure (DBP), while CMT only elevated SBP [5]. Unlike La Monica et al. (2021) and Tartar et al. (2021), where the caffeine content was matched to the caffeine in CMT, Cintineo et al. (2022) doubled the caffeine content as compared to CMT in the caffeine condition. Given the differences between caffeine and CMT shown in these previous investigations, there are theoretical benefits to TeaCrine^®^ and methylliberine that should be explored further.

The pharmacokinetics of methylliberine, TeaCrine^®^, and caffeine have been compared. A 100 mg dose of methylliberine showed peak plasma concentrations at 0.8–0.9 h and a half-life of 1.4–1.5 h [13,14]. Although the half-lives of caffeine and TeaCrine^®^ differ depending on the dose, TeaCrine^®^ has been shown to have a much longer half-life than caffeine [15] and both have extended half-lives compared to methylliberine [14]. Methylliberine has been shown to reduce the oral clearance and extend the half-life of caffeine when co-administered, however, caffeine does not appear to affect the pharmacokinetics of methylliberine, at least at a 100 mg dose [13]. The conclusion is that caffeine and methylliberine likely differ in their affinity and selectivity for adenosine A_1_ and A_2A_ receptors, which may also result in different effects [13,16,17]. Currently, there have not been any studies conducted on the effects of independent methylliberine ingestion on cognitive function or indices of affect. Within the two studies that have investigated the independent ingestion of methylliberine, neither one reported any influence on vital signs, respiratory rate, body temperature, or mood in men and women [8], nor did it negatively affect markers of health [9]. Thus, the purpose of this study was to assess the acute and potential accumulative effects of methylliberine supplementation on cognitive function and overall well-being, including energy, sustained energy, mental stamina, focus, concentration, motivation to accomplish difficult tasks, drive, vigor, positive outlook, maintaining a healthy mood, feelings of well-being, and resilience to stress. Our hypothesis was that methylliberine ingestion would improve cognitive function, energy, mood, and focus without negatively impacting heart rate or blood pressure.

## 2. Methods

### 2.1. Experimental Design

This was a double-blind, randomized, two-arm, within-subject crossover trial in which participants visited the laboratory on five occasions (one screening visit, two baseline visits, and two post-supplement testing visits). This study was conducted according to the guidelines laid down in the Declaration of Helsinki of 1975 and all procedures involving human subjects were approved by the Genetic Alliance IRB on 9/30/22 (#CSI-08-2022-001). Written informed consent was obtained from all subjects prior to enrollment. The study was registered on clinicaltrials.gov (“Effects of Dynamine Ingestion on Various Indices of Sustained Energy”, #NCT06048640). This study was conducted at a contract research organization (CRO) in Northeast Ohio. During the initial screening visit, each participant’s medical history and blood work (CBC, CMP, and lipid panel) were assessed, their baseline diet was evaluated, and each participant underwent 3 sets of familiarization trials of the neuropsychological assessments used within our testing procedures (Stroop and Trail Making Test B (TMT-B)). Since this study employed a crossover design, there were two baseline visits (one for each product tested). Thus, during the baseline visits (visits 2 and 4, which were prior to the supplementation administration), the subjects completed baseline testing which included subjective questionnaires (visual analog scales (VAS)) that assessed energy, sustained energy, mental stamina, focus, concentration, motivation to accomplish difficult tasks, drive, vigor, positive outlook, maintaining a healthy mood, feelings of well-being, and resilience to stress, in addition to completing 3 sets of neuropsychological assessments (Stroop and TMT-B) to assess their mental processing, cognitive flexibility, and attention. During visits 2 and 4, the participants completed the neuropsychological assessments twice with 10 min in between. At the conclusion of visits 2 and 4, the participants were given their respective supplements (i.e., placebo or Dynamine™) to take for three days and return on the fourth day. The participants returned for visits 3 and 5, where they took a fourth dose of their respective supplements at the laboratory and repeated the same testing procedures outlined at their baseline visits (visits 2 and 4), except they were administered at four timepoints (prior to ingestion of the 4th dose, 1 h, 2 h, and 3 h post-ingestion). There were no less than 7 days in between the completion of one investigational product and the start of the second investigational product (i.e., in between visits 3 and 4). For example, if a subject received the placebo first and Dynamine™ second, then visit 2 would be the baseline testing day for the placebo and visit 3 would coincide with the 4th consecutive dose for the placebo. The 4th dose of the placebo would be administered after the first round of testing (baseline/pre) on visit 3. There would then be at least 7 days before the participant would come back for a second baseline testing day (visit 4) for Dynamine™, and then come back a final time for the fourth consecutive dose of Dynamine™ (visit 5) to undergo the same testing procedures as in visit 3.

### 2.2. Participants

In total, 25 healthy men and women completed all study visits (see Table 1 for subject characteristics). All the participants were in good health, as determined by physical examination and medical history, between the ages of 21 and 55 years, and had a body mass index (BMI) of 18.5–27 kg·m^−2^. Prior to participation, all the participants indicated their willingness to comply with all aspects of the experimental and supplement protocol. Participants were excluded if they: (a) had a history of diabetes or pre-diabetes; (b) had a history of malignancy in the previous 5 years, except for non-melanoma skin cancer (basal cell cancer or squamous cell cancer of the skin); (c) had prior gastrointestinal bypass surgery; (d) had known gastrointestinal or metabolic diseases that might impact their nutrient absorption or metabolism (e.g., short bowel syndrome, diarrheal illnesses, history of colon resection, gastro paresis, and Inborn-Errors-of-Metabolism); (e) had any chronic inflammatory condition or disease; (f) had a known allergy to any of the ingredients in the supplement or the placebo; (g) were currently been participating in another research study with an investigational product or had been in another research study in the past 30 days; (h) had a caffeine intake of three or more cups of coffee or equivalent (>400 mg) per day; (i) used corticosteroids or testosterone replacement therapy (ingestion, injection, or transdermal); (j) had any other diseases or conditions that, in the opinion of the medical staff, could confound the primary endpoint or place the participant at an increased risk of harm if they were to participate; or (k) did not demonstrate a verbal understanding of the informed consent document.

The participants were instructed to follow their normal diet and activity patterns throughout their participation in the study. The participants were required to complete a 24 h diet record prior to arriving at the laboratory for their initial screening visit. The participants were given a copy of this dietary record and instructed to duplicate all food and fluid intake 24 h prior to each subsequent laboratory visit. Prior to each subsequent visit, the participants were asked to verbally confirm their 24 h prior diet adherence and ensure they had a normal night’s rest. In addition to replicating food and fluid intake for 24 h prior, the study participants were also asked to refrain from exercise and alcohol 24 h prior, abstain from caffeine 12 h prior, and arrive 8 h fasted to all testing sessions. Again, these instructions were all verbally confirmed at the beginning of each study visit.

### 2.3. Neuropsychological Assessments

The Stroop test measures the ability to inhibit cognitive interference, attention, processing speed, cognitive flexibility [18], and working memory [19]. The Stroop test requires individuals to read color words printed in a different color ink (for example, the word “green” could be printed in blue) and select the color of the ink they see instead of reading the word they see (therefore, within the example, the answer would be blue) [20]. This challenge requires participants to perform a less automated task (i.e., naming the ink color) while inhibiting the interference coming from a more automated task (i.e., reading the word) [20]. All the participants in the study were assessed using the congruent standard condition of the Stroop test for a duration of two minutes at each attempt/repetition. The outcome variables associated with the Stroop test were total score, accuracy, and average time per score, which were all calculated and provided by the testing application (Andrew Novak Stroop Test for Research App).

The TMT-B requires individuals to connect 25 encircled numbers and letters in numerical and alphabetical order [21]. For example, the number “1” is followed by “A”, which is then followed by “2” then “B” and so forth and so on [21]. The outcome variable associated with TMT-B was time to completion, which was calculated and provided by the testing application (version 1.2). TMT-B measures cognitive flexibility with its visually interfering stimuli and the physical distance between numbers/letters [21].

The participants completed the Stroop test and TMT-B with one minute of rest in between each test and each set. In total, the participants underwent 3 sets of Stroop and TMT-B tests at each time point. The median value was taken as the value for each respective time point. During baseline visits 2 and 4, the participants completed the 3 sets of the Stroop and TMT-B tests twice with a 10 min break in between. The two median values from these two timepoints in visits 2 and 4 were averaged and used in the statistical analyses as the participants’ baseline. During visits 3 and 5, the participants completed the 3 sets of the Stroop and TMT-B tests at baseline/pre (before supplement ingestion), 1 h, 2 h, and 3 h post-ingestion of their respective supplements.

### 2.4. Visual-Analog Scales

The participants completed 100 mm anchored VASs before and after baseline testing, 1 h, 2 h, and 3 h after the ingestion of each supplement on visits 3 and 5, and twice on visits 2 and 4 (before and after their first cognitive testing session). The VASs were anchored with “Not very focused”, “Very low initiative”, or “Lowest Possible” and “Highly focused”, “Very high initiative” or “Highest Possible” and assessed subjective ratings of energy, sustained energy, mental stamina, ability to focus, ability to concentrate, motivation to accomplish difficult tasks, drive, vigor, positive outlook, mood, feeling of well-being, and ability to tolerate stress. Again, the two values (before and after the first cognitive testing session) for each subjective rating on visits 2 and 4 were averaged and then used in the statistical analyses as the participants’ baseline. The validity and reliability of the VAS for assessing fatigue and energy have been previously established [22] and reported [23,24].

### 2.5. Supplement Protocol

Throughout the study protocol, all the supplements were prepared in single capsule form for oral ingestion and packaged in coded generic containers for administration. The participants orally ingested PLA (100 mg cellulose) and 100 mg of methylliberine (as Dynamine™). Using a cross-over design, half of the subjects were randomly assigned to receive the PLA first, while the other half were assigned to receive the methylliberine (Dynamine™) first. At the conclusion of visits 2 and 4, the participants were given a packet of three daily doses (1 dose/day) to consume prior to visits 3 and 5, respectively. The fourth dose was consumed in the laboratory after their baseline/pretesting timepoint (which included cognitive testing and VAS) in the presence of the medical staff. There were no less than 7 days in between each trial (i.e., in between visit 3 and 4).

### 2.6. Anthropometric and Other Resting Measures

Standing height was determined using a wall-mounted stadiometer and body weight was measured using a Seca 767TM Medical Scale (body weight was measured at each visit). Resting heart rate and blood pressure were measured using an automated blood pressure cuff (Omron HEM-780) before and after each timepoint (i.e., baseline, 1 h, 2 h, and 3 h post-ingestion of each assigned supplement) during visits 3 and 5. Similarly, resting heart rate and blood pressure were measured before and after the first testing session during visits 2 and 4.

### 2.7. Adverse Events (AEs)

All adverse events (all local and systemic non-serious and serious) were monitored by the researchers and evaluated and assessed through reports coded using the Medical Dictionary for Regulatory Activities (MedDRA). In the event of an AE, the intensity of the AE would be graded according to the protocol-defined criteria based on the Common Terminology Criteria for Adverse Events (CTCAE) Version 5.0, 2017.

### 2.8. Statistical Analyses

The primary outcome measures included cognitive focus and attention (i.e., total score, accuracy, average time per score, and time to completion), determined by neuropsychological testing (Stroop and TMT-B) to assess objective changes in mental processing, cognitive flexibility, and attention, with VASs assessing subjective changes in energy, sustained energy, mental stamina, focus, concentration, motivation to accomplish difficult tasks, drive, vigor, positive outlook, mood, feelings of well-being, and resilience to stress. The secondary outcome measures included vital signs (blood pressure and heart rate) and side effect profile/adverse events monitoring. Data are presented as means ± standard deviation and the primary statistical approach employed was a mixed factorial ANOVA with repeated measures on time to assess group (methylliberine vs. PLA), time, and group × time interaction effects. The primary analysis compared the acute effects at all timepoints within visits 3 and 5, while the secondary analysis compared baseline (values that were averaged) at visits 2 and 4 to the baseline/pre timepoint (prior to the 4th dose ingestion of the study product) at visits 3 and 5 (examining whether an accumulation over 3 daily doses had any effect on the primary outcomes). An a priori power analysis was conducted for the primary analyses’ main outcome measures using G*Power (i.e., specifically energy). The results for the mixed factorial ANOVA with repeated measures, with two groups and four time points, within-between interaction, and a small effect of 0.25, was a sample size of 24 to achieve 80% power. All the variables were tested for normality using the results from a Shapiro–Wilk test. When a deviation from normality was identified, natural log transformations were employed. As such, transformations were employed for the Stroop accuracy scores. In the case of scale (ratio) data, transformations were not possible. Factorial ANOVAs with repeated measures on time were used to examine changes from baseline (the first testing session on visits 3 and 5) within each group, with Bonferroni corrections applied to all pairwise comparisons. Changes from baseline (deltas) were calculated and independent *t*-tests were computed to evaluate the between-group changes using 95% confidence intervals, *p*-values, and effect sizes. Non-normal data were first analyzed using the Friedman test (within-group changes), then the Wilcoxon signed rank test (paired differences within group), and then Mann–Whitney U test (between-group differences). A significance level of 0.05 was used for all statistical determinations, while *p*-values between 0.051 and 0.10 were deemed a trend. All the statistical analyses were conducted using SPSS version 23.

## 3. Results

In total, 13 women and 12 men completed all the study visits. See Table 1.

### 3.1. Stroop

There were no differences in the total scores between the baseline visit and after 3 days of supplementation (group: *p* = 0.61; time: *p* = 0.44; and group × time: *p* = 0.29). However, there was an acute improvement over time after the fourth dose, regardless of group (group: *p* = 0.21; time: *p* < 0.001; and group × time: *p* = 0.61).

There were no differences in accuracy between the baseline visit and after 3 days of supplementation (group: *p* = 0.440; time: *p* = 0.850; and group × time: *p* = 0.99). There were no acute differences in accuracy after the fourth dose (group: *p* = 0.56; time: *p* = 0.42; and group × time: *p* = 0.93).

There were no differences in average time per score between the baseline visit and after 3 days of supplementation (group: *p* = 0.45; time: *p* = 0.39; and group × time: *p* = 0.45). However, there was an acute improvement over time after the fourth dose, regardless of group (group: *p* = 0.22; time: *p* < 0.001; and group × time: *p* = 0.63). See Table 2.

### 3.2. Trail Making Test B (TMT-B)

There was a trend for the group × time interaction and a significant main effect of time for time to completion between the baseline visit and after 3 days of supplementation (group: *p* = 0.56; time: *p* = 0.001; and group × time: *p* = 0.06). Post hoc analyses showed that the time to completion after 3 days of supplementation was ~11% lower (i.e., improved) vs. the baseline visit for methylliberine (*p* < 0.001). Additionally, there was an acute improvement over time after the fourth dose, regardless of group (group: *p* = 0.65; time: *p* = 0.009; and group × time: *p* = 0.13). See Table 2.

### 3.3. Visual Analog Scales (VAS)

**Figure 1:**
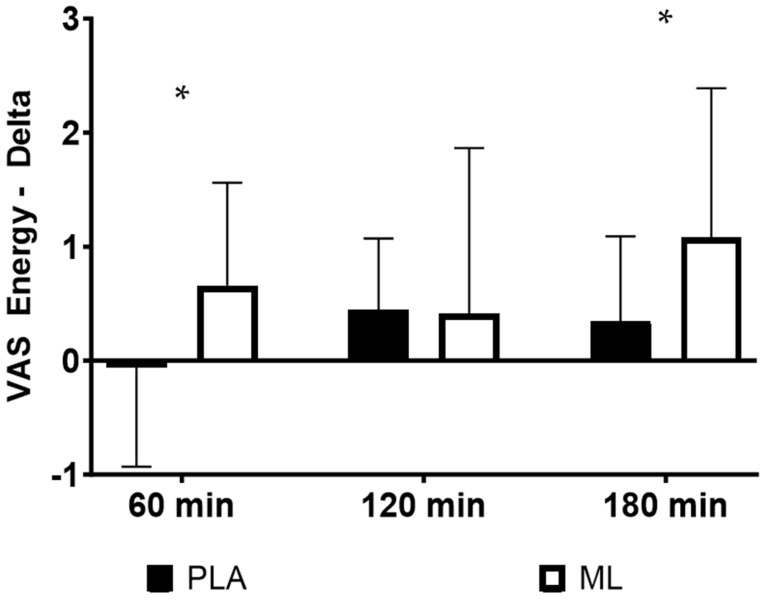
The change in energy relative to the baseline time point (0 min) for women between groups. * Significantly different from PLA (*p* ≤ 0.05). Data are shown as Mean and SD.

There were no differences in the subjective ratings of energy between the baseline visit and after 3 days of supplementation (group: *p* = 0.84; time: *p* = 0.26; and group × time: *p* = 0.27). However, there was an acute improvement over time, regardless of group, after the fourth dose (group: *p* = 0.14; time: *p* = 0.001; and group × time: *p* = 0.23). Additionally, there were no differences in the deltas (*p* > 0.050) from the baseline timepoint after the fourth dose for energy (See Table 3). There was a trend for the group × sex × time interaction (*p* = 0.064), showing that the two sexes may have responded differently in terms of energy between the two treatment conditions over time. A post hoc 2 × 4 (group × time) mixed factorial ANOVA with repeated measures showed that women had a significant group × time interaction (*p* = 0.020), while the men did not (*p* = 0.810). Post hoc testing on the deltas showed that women had a greater change in energy from the baseline timepoint (0 min) to 1 h post-supplementation (*p* = 0.010, 95%CI: 0.21 to 1.23, d = 0.81) and from the baseline timepoint to 3 h post-supplementation (*p* = 0.040, 95%CI: 0.06 to 1.40, d = 0.69) in methylliberine vs. PLA. See Figure 1.

**Figure 2:**
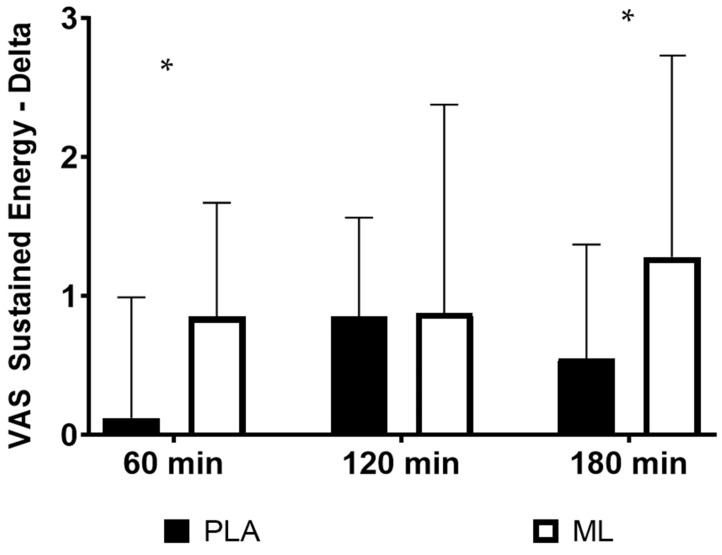
The change in sustained energy relative to the baseline time point (0 min) for women between groups. * Significantly different from PLA (*p* ≤ 0.05). Data are shown as Mean and SD.

There were no differences in the subjective ratings of sustained energy between the baseline visit and after 3 days of supplementation (group: *p* = 0.75; time: *p* = 0.97; and group × time: *p* = 0.38). However, there was an acute improvement over time, regardless of group, after the fourth dose (group: *p* = 0.24; time: *p* < 0.001; and group × time: *p* = 0.33). Additionally, there were no differences in the deltas (*p* > 0.050) from the baseline timepoint after the fourth dose for sustained energy (See Table 3). There was a significant group × sex × time interaction (*p* = 0.034), showing that the two sexes responded differently in terms of sustained energy between the two treatment conditions over time. A post hoc 2 × 4 (group × time) mixed factorial ANOVA with repeated measures showed that women had a significant group × time interaction (*p* = 0.022), while the men did not (*p* = 0.68). Post hoc testing on the deltas showed that women had a greater change in sustained energy from the baseline timepoint (0 min) to 1 h post-supplementation (*p* = 0.030, 95%CI: 0.10 to 1.38, d = 0.86) and from the baseline timepoint (0 min) to 3 h post-supplementation (*p* = 0.040, 95%CI: 0.04 to 1.41, d = 0.62) in methylliberine vs. PLA. See Figure 2.

There were no differences in the subjective ratings of mental stamina between the baseline visit and after 3 days of supplementation (group: *p* = 0.32; time: *p* = 0.74; and group × time: *p* = 0.11). However, there was an acute improvement over time, regardless of group, after the fourth dose (group: *p* = 0.15; time: *p* = 0.003; and group × time: *p* = 0.41). Additionally, there were no differences in the deltas (*p* > 0.050) from the baseline timepoint (0 min) after the fourth dose for mental stamina. See Table 3.

There were no differences in the subjective ratings of focus between the baseline visit and after 3 days of supplementation (group: *p* = 0.61; time: *p* = 0.58; and group × time: *p* = 0.91). However, there was an acute improvement over time, regardless of group, after the fourth dose (group: *p* = 0.18; time: *p* = 0.03; and group × time: *p* = 0.33). There was a trend for a difference in the deltas from the baseline timepoint (0 min) to 3 h post-supplementation, showing that methylliberine may have had a greater increase in focus relative to baseline vs. PLA (*p* = 0.08, mean difference = 0.44 ± 0.24 cm, 95%CI: −0.06 to 0.93, d = 0.38). See Table 3.

**Figure 3:**
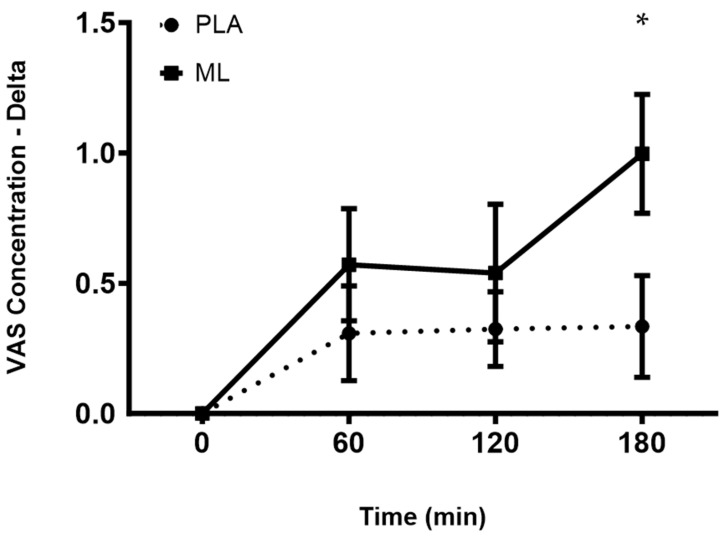
The change in concentration relative to the baseline time point (0 min) between groups. PLA: Placebo. ML: Methylliberine. * Significantly different from PLA (*p* ≤ 0.05). Data are shown as Mean and SD.

There were no differences in the subjective ratings of concentration between the baseline visit and after 3 days of supplementation (group: *p* = 0.89; time: *p* = 0.91; and group × time: *p* = 0.98). There was a group × time interaction and a significant time effect after the fourth dose (group: *p* = 0.17; time: *p* = 0.002 and group × time: *p* = 0.03). A post hoc analysis showed improvements in concentration over time for methylliberine, but not for PLA. Specifically, for methylliberine, 1 h (~10.2%, *p* = 0.045) and 3 h (~15.3%, *p* = 0.004) post-supplementation were significantly greater than the baseline time point (0 min). Also, the delta from baseline (0 min) to 3 h post-supplementation was significantly larger for methylliberine vs. PLA (*p* = 0.006, mean difference = 0.70 ± 0.23 cm, 95%CI: 0.22 to 1.18, d = 0.68). See Figure 3.

**Figure 4:**
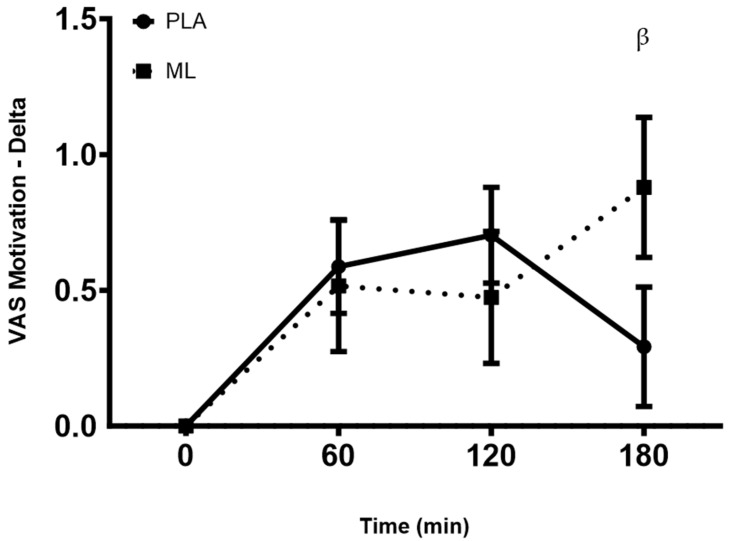
The change in motivation relative to the baseline time point (0 min) between groups. PLA: Placebo. ML: Methylliberine. ^β^ Statistical trend from PLA (*p* ≤ 0.1). Data are shown as Mean and SD.

There were no differences in the subjective ratings of motivation between the baseline visit and after 3 days of supplementation (group: *p* = 0.45; time: *p* = 0.29; and group × time: *p* = 0.63). There was a group × time interaction and a significant time effect after the fourth dose (group: *p* = 0.19; time: *p* < 0.001, and group × time: *p* = 0.02). A post hoc analysis showed greater motivation at 1 h (~10.9%, *p* = 0.010) and 2 h (~12.7%, *p* = 0.003) post-supplementation vs. baseline (0 min) for PLA, and greater motivation at 3 h post-supplementation vs. baseline (0 min) for methylliberine (~15.8%, *p* = 0.004). Also, the delta from baseline (0 min) to 3 h post-supplementation may have been larger for methylliberine vs. PLA (*p* = 0.06, mean difference = 0.53 ± 0.27 cm, 95%CI: −0.02 to 1.08, d = 0.50). See Figure 4.

There were no differences in the subjective ratings of drive between the baseline visit and after 3 days of supplementation (group: *p* = 0.37; time: *p* = 0.65; and group × time: *p* = 0.97). However, there was a positive impact over time, regardless of group, after the fourth dose (group: *p* = 0.45; time: *p* = 0.02; and group × time: *p* = 0.36). Additionally, there were no differences in the deltas (*p* > 0.050) from the baseline timepoint (0 min) after the fourth dose for drive. See Table 3.

There were no differences in the subjective ratings of vigor between the baseline visit and after 3 days of supplementation (group: *p* = 0.83; time: *p* = 0.97; and group × time: *p* = 0.78). However, there was a trend for the group × time interaction and a significant time effect after the fourth dose (group: *p* = 0.470; time: *p* = 0.002; and group × time: *p* = 0.10). A post hoc analysis showed greater vigor 2 h post-supplementation vs. baseline (0 min) for PLA (~10.5%, *p* < 0. 009) and greater vigor 3 h post-supplementation vs. baseline (0 min) for methylliberine (~10.3%, *p* = 0.009). Additionally, there were no differences in the deltas (*p* > 0.050) from the baseline timepoint (0 min) after the fourth dose for vigor. See Table 3.

There was a significant main effect of time between the baseline visit and after 3 days of supplementation for positivity (group: *p* = 0.64; time: *p* = 0.01; and group × time: *p* = 0.62), which showed a decrease in the subjective ratings of positive outlook, regardless of group. There was a trend for the group × time interaction and a significant time effect (group: *p* = 0.62; time: *p* < 0.001; and group × time: *p* = 0.08) after the fourth dose, showing an increase in positive outlook regardless of group. A post hoc analysis showed greater positivity 2 h post-supplementation vs. baseline (0 min) for PLA (~9.4%, *p* < 0.001) and greater positivity 3 h post-supplementation vs. baseline (0 min) for methylliberine (~9.4%, *p* = 0.002). There was a trend for a delta, showing positivity increasing to a greater degree from baseline (0 min) to 3 h post-supplementation for methylliberine vs. PLA (*p* = 0.100, mean difference = 0.46 ± 0.27 cm, 95%CI: −0.09 to 1.01, d = 0.43). See Table 3.

**Figure 5:**
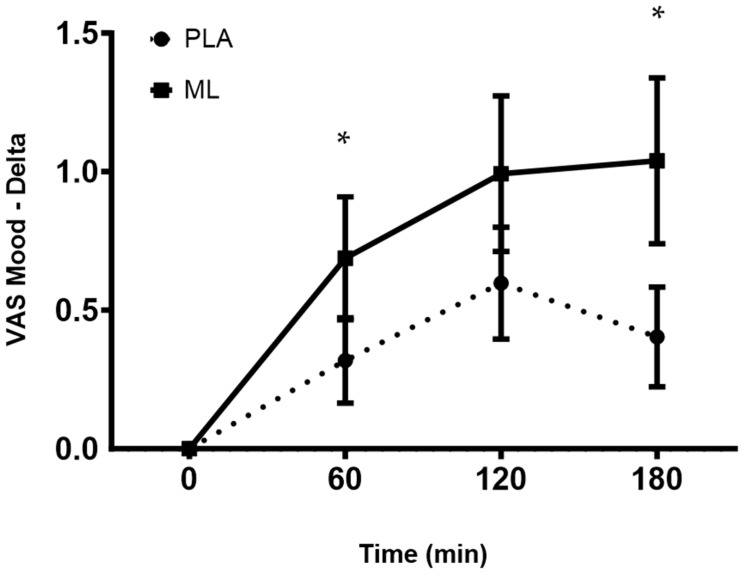
The change in mood relative to the baseline time point (0 min) between groups. PLA: Placebo. * Significantly different from PLA (*p* ≤ 0.05). Data are shown as Mean and SD.

There was a significant main effect of time between the baseline visit and after 3 days of supplementation for mood (group: *p* = 0.54; time: *p* = 0.01; and group × time: *p* = 0.70), which showed a decrease in the subjective ratings of mood, regardless of group. There was a group × time interaction and a significant time effect (group: *p* = 0.48; time: *p* < 0.001; and group × time: *p* = 0.04) after the fourth dose. A post hoc analysis showed a more positive mood at 1 h (~9.8%, *p* = 0.020), 2 h (~14.8%, *p* = 0.004), and 3 h (~16.4%, *p* = 0.004) post-supplementation vs. baseline (0 min) for methylliberine and at 2 h post-supplementation vs. baseline (0 min) for PLA (~9.7%, *p* = 0.047). Also, the delta from baseline (0 min) to 1 h post-supplementation (*p* = 0.05, mean difference = 0.38 ± 0.18 cm, 95%CI: −0.01 to 0.76, d = 0.43) and the delta from baseline (0 min) to 3 h post-supplementation (*p* = 0.03, mean difference = 0.72 ± 0.30 cm, 95%CI: 1.35 to 2.37, d = 0.65) were significantly greater for methylliberine vs. PLA, showing that methylliberine improved mood to a greater degree at 1 h and 3 h post-ingestion. See Figure 5.

There was a significant main effect of time between the baseline visit and after 3 days of supplementation for well-being (group: *p* = 0.15; time: *p* = 0.01; and group × time: *p* = 0.92), which showed a decrease in the subjective ratings of well-being, regardless of group. There was a trend for the group × time interaction and a significant time effect (group: *p* = 0.75; time: *p* < 0.001; and group × time: *p* = 0.09) after the fourth dose. A post hoc analysis showed a more positive rating for well-being at 2 h (~14.1%, *p* = 0.013) and 3 h (12.5%, *p* = 0.027) post-supplementation vs. baseline (0 min) for methylliberine. Also, the delta from baseline (0 min) to 3 h post-supplemenrtation (*p* = 0.050, mean difference = 0.54 ± 1.34 cm, 95%CI: −0.01 to 1.10, d = 0.52) was significantly greater for methylliberine vs. PLA, showing that well-being improved to a greater degree with methylliberine. See Table 3.

There was a significant main effect of time between the baseline visit and after 3 days of supplementation for the ability to tolerate stress (group: *p* = 0.89; time: *p* = 0.002; and group × time: *p* = 0.90), which showed a decrease in the ability to tolerate stress, regardless of group. There was a trend for the group × time interaction and a significant time effect (group: *p* = 0.29; time: *p* = 0.03; and group × time: *p* = 0.08). A post hoc analysis showed a more positive rating for the ability to tolerate stress at 2 h post-supplementation vs. baseline (0 min) for methylliberine (~12.3%, *p* = 0.010). Also, the delta from baseline (0 min) to 3 h post-supplementation (*p* = 0.040, mean difference = 0.67 ± 0.30 cm, 95%CI: 0.04 to 1.29, d = 0.54) was significantly greater for methylliberine vs. PLA, showing that the ability to tolerate stress was improved to a greater degree with methylliberine. See Table 3.

### 3.4. Hemodynamics

There were no differences in heart rate levels between the baseline visit and after 3 days of supplementation (group: *p* = 0.38; time: *p* = 0. 59; and group × time: *p* = 0.58). There was a significant time effect (group: *p* = 0.501; time: *p* < 0.001; and group × time: *p* = 0.421) after the fourth dose, showing a decrease in heart rate, regardless of group. There were no differences in the deltas between groups at 60 min (*p* = 0.250), 120 min (*p* = 0.920), and 180 min (*p* = 0.230) post-ingestion. See Table 4.

There was a significant condition effect for the systolic blood pressure levels between the baseline visit and after 3 days of supplementation (group: *p* = 0.03; time: *p* = 0.72; and group × time: *p* = 0.13). There were no acute differences after the fourth dose (group: *p* = 0.914; time: *p* = 0.101; and group × time: *p* = 0.495). There were no differences in the deltas between groups at 60 min (*p* = 0.660), 120 min (*p* = 0.790), and 180 min (*p* = 0.300) post-ingestion. See Table 4.

There were no differences in the diastolic blood pressure levels between the baseline visit and after 3 days of supplementation (group: *p* = 0.55; time: *p* = 0. 56; and group × time: *p* = 0.25). When acute changes across time were evaluated in response to the day 4 supplementation, a group × time interaction (*p* = 0.004) was present, while the main effects for time (*p* = 0.367) and condition were not significant (*p* = 0.624). A post hoc analysis using independent *t*-tests of the observed changes from baseline (0 min) revealed no significant differences between groups after 60 min (*p* = 0.450) and 180 min (*p* = 0.120). However, a significantly greater diastolic blood pressure was observed with PLA vs. methylliberine after 120 min (*p* = 0.001, mean difference = 5.84 ± 1.55 mmHg, 95% CI: 2.63, 9.05 mmHg, d = −0.84). See Table 4.

## 4. Discussion

This investigation sought to examine the effect of the ingestion of methylliberine on cognitive performance and subjective feelings of well-being. The acute ingestion of methylliberine improved concentration, motivation, and mood more profoundly and sustained positive effects for longer than PLA. Furthermore, the acute ingestion of methylliberine may have improved well-being and the ability to tolerate stress, while the short-term supplementation of methylliberine (i.e., after three daily doses) may have improved time to completion on the TMT-B test. On the other hand, there were no acute differences in the cognitive performance measures between PLA and methylliberine. Interestingly, methylliberine had a positive impact on energy and sustained energy for women as compared to men, which may have been driven by differences in body size/weight. There were negligible differences between treatments in terms of vital signs, apart from a higher diastolic blood pressure 2 h post-ingestion during the PLA treatment. Lastly, both treatments were very well-tolerated, without any adverse events reported.

To our knowledge, this is the first study evaluating the impact of the independent ingestion of methylliberine on common cognitive function tests. The Stroop test measures working memory and attention control [19], while the TMT-B measures processing speed, sequencing, mental flexibility, and visual–motor skills [21]. Consistent with PLA, methylliberine did not significantly impact the neuropsychological assessments performed in this study (i.e., the Stroop test and TMT-B). Neither treatment improved the Stroop test total score, accuracy, or reaction time, However, there was a potential ~11% improvement in the TMT-B time to completion after three daily doses of methylliberine. This may suggest that longer periods of methylliberine use may be needed to optimize cognitive enhancements. Comparatively, the independent ingestion of TeaCrine^®^ (a closely related methylurate) was not able to improve the cognitive measures of performance against PLA during a simulated soccer match [25], however, caffeine was shown to decrease the reaction times in a Stroop test interspersed with intermittent exercise in soccer players [26]. Meanwhile, the combination of CMT has been reported to improve cognitive performance after gaming and go/no go tasks [5,6,7]. These findings imply an additive/synergistic effect of combining caffeine (a methylxanthine) with Methylliberine and TeaCrine^®^ (which are methylurates).

Methylliberine had a significant positive impact on mood, motivation, and concentration. Notably, an acute 100 mg dose of methylliberine uniquely improved concentration by approximately 10.2% one-hour post-ingestion and approximately 15.3% three hours post-ingestion. Although PLA improved motivation 1 and 2 h post-ingestion and mood 2 h post-ingestion, methylliberine increased motivation 3 h post-ingestion and improved mood 1, 2, and 3 h post-ingestion. Methylliberine also had a larger positive change in mood from baseline to 1 h and 3 h post-ingestion. The consistent improvements in methylliberine at the 3 h mark were notable and unexpected given its short half-life of 1.5 ± 0.8 h [14]. Other potential benefits observed with methylliberine included a more positive state of well-being 2 and 3 h post-ingestion, an enhanced ability to tolerate stress 2 h post-ingestion, and greater vigor and positivity 3 h post-ingestion. In opposition, a previous investigation did not report any influence after an acute 100 mg dose of methylliberine on attentiveness, energy, motivation, irritability, focus, and mood [8]. However, the study treatments in the previous investigation [8] did not include a PLA treatment for comparison; therefore, the impact methylliberine had on subjective feelings of affect was not definitive. A previous pilot study employing 100 mg and 150 mg doses of methylliberine alone or in conjunction with TeaCrine^®^ vs. a PLA observed significant main effects of time for alertness, productivity, and motivation to perform mental tasks after 1, 2, and 4 weeks of supplementation [27]. However, in addition to having a longer supplementation period than the current study, the results of Stratton et al. (2018) provide vague differences over time regardless of group and do not indicate a positive or negative influence from either investigational product. In comparison, there have been mixed results observed with an acute dose of TeaCrine^®^, showing increased energy, reduced fatigue, and possibly improved concentration [28], while an 8-week dose regimen increased vigor (via POMS), but had negligible effects on focus, concentration, and energy [29]. Meanwhile, the acute ingestion of CMT did not impact energy, alertness, focus, creativity, or decision making compared to PLA and caffeine [6], but positively impacted mood compared to PLA in egamers [7]. Also, CMT did not differ from caffeine, TeaCrine^®^, or methylliberine alone or compared to combinations of TeaCrine^®^ + methylliberine or methylliberine + caffeine in terms of subjective energy, attentiveness, motivation, focus, or moodiness over a 48 h period [8]. Therefore, this is the first study demonstrating the beneficial effects of methylliberine ingestion alone on several indices of overall well-being.

An interesting observation in the current investigation was the notable increases in energy and sustained energy that were unique to women. After an acute dose of methylliberine, women had a greater increase in sustained energy and a potential increase in energy 1 and 3 h post-ingestion relative to baseline vs. PLA. Similarly, Taylor et al. (2016) noted increased levels of vigor from week 4 to week 8 with daily TeaCrine^®^ ingestion that were unique to women. Previous observations on caffeine have observed sex-specific impacts in terms of physical performance [30] and memory [31], potentially mediated by sex hormones’ influence on caffeine metabolism [32]. Meanwhile, there have not been any reported sex-specific differences with CMT consumption. The observed differences in the current investigation could be due to men having a larger frame and greater body weight than women. On average, men ingested 1.27 mg/kg and women ingested 1.47 mg/kg of methylliberine. Future research on methylliberine may need to consider dosing relative to body weight, similar to how caffeine recommendations are often structured based on body weight [33].

Methylliberine had minimal effects on vitals. In fact, an increase in DBP was noted 2 h after PLA ingestion, while there were no changes over time with methylliberine. Therefore, the current investigation corroborates previous findings showing no effect on hemodynamics with methylliberine ingestion [8,9]. Likewise, TeaCrine^®^ did not impact hemodynamics over 8 weeks [29] or in combination with methylliberine [9] under acute or chronic conditions. An investigation assessing methylliberine, TeaCrine^®^, caffeine, and various combinations of the three observed higher blood pressure only when caffeine was included in the treatment [8]. However, it should be noted that the observed increases were small, transient, and within normal clinical limits.

Given that caffeine and TeaCrine^®^ both act as adenosine receptor antagonists and increase dopamine transmission [34], which can impact behavioral activation and effort-related processes [35,36], one would expect caffeine, TeaCrine^®^, and methylliberine to have similar physiological and performance impacts, however, previous research [8,9] and the current investigation have shown otherwise. Our primary analysis of the acute effects had an adequate sample size for statistical power, however, our secondary analysis examining the effects of a three-dose regimen was slightly underpowered, perhaps explaining the observed findings. Unfortunately, due to the limited scope of research on the novel compound (i.e., methylliberine), we cannot mechanistically deduce the observed effects. Therefore, additional studies on methylliberine should be undertaken to explore the potential of this unique compound. Given the observed differences in women with perceptions of energy status, future studies should also investigate sex effects and doses relative to body weight. Nonetheless, methylliberine may offer a viable, non-habitual alternative for those sensitive to caffeine and looking for a positive impact on their well-being.

Our study demonstrated that methylliberine did not enhance cognitive function, but did significantly enhance subjects’ perceptions of energy, concentration, motivation, and mood over time. Methylliberine also improved concentration, well-being, and the ability to tolerate stress to a greater degree than PLA, while having no detrimental effects on vital signs (blood pressure and heart rate). Methylliberine also seems to have had a positive impact on the self-reported ratings of sustained energy in women. Although the placebo improved motivation and mood post-ingestion (at hours 1 and 2), methylliberine sustained these benefits for longer (up to 3 h post-ingestion) on the acute testing day (i.e., the fourth dose).

## Figures and Tables

**Table 1:** Participant characteristics.

	Men (N = 12)	Women (N = 13)
Age (years)	33.5 ± 10.7	33.5 ± 11.1
Height (cm)	180.6 ± 7.7	170.8 ± 7.2
Weight (kg)	79.0 ± 8.0	68.1 ± 11.1
Body Mass Index (kg/m^2^)	24.9 ± 1.4	23.9 ± 2.6
Systolic Blood Pressure (mm Hg)	122.4 ± 8.7	113.3 ± 13.5
Diastolic Blood Pressure (mm Hg)	78.6 ± 9.8	76.5 ± 7.8
Resting Heart Rate (bpm)	66.2 ± 8.7	70.0 ± 12.6

**Table 2:** Stroop and Trail Making Test B (TMT-B) scores at baseline (Day 0) and testing visits (Day 4).

	Stroop Test						TMT-B	
	Total Score (au)		Accuracy (%)		Average Time per Score (ms)		Time to Completion (s)	
Time	PLA	ML	PLA	ML	PLA	ML	PLA	ML
Day 0	125.2 ± 18.4	125.0 ± 17.9	99.1 ± 1.2	99.2 ± 1.1	0.99 ± 0.14	0.98 ± 0.13	22.8 ± 6.1	24.3 ± 7.2
0 min (Day 4)	124.7 ± 19.2	127.4 ± 18.8	99.0 ± 1.1	99.2 ± 1.3	0.99 ± 0.17	0.96 ± 0.14	21.8 ± 5.5	21.6 ± 5.8 ^⸸^
60 min (Day 4)	128.5 ± 17.4	131.5 ± 17.5 *	99.0 ± 1.3	99.1 ± 1.8	0.95 ± 0.14	0.93 ± 0.12	21.7 ± 6.0	20.4 ± 5.2
120 min (Day 4)	131.3 ± 17.0 *	132.1 ± 16.3 *	98.8 ± 1.7	99.0 ± 1.2	0.93 ± 0.12	0.92 ± 0.11 *	21.1 ± 5.4	20.2 ± 4.1
180 min (Day 4)	131.7 ± 17.7	134.8 ± 17.7 *	98.8 ± 1.3	98.9 ± 1.8	0.92 ± 0.12 *	0.91 ± 0.11 *	19.3 ± 4.0 *	20.3 ± 3.8

PLA: Placebo. ML: Methylliberine. * Statistically significant difference from 0 min time point (*p* ≤ 0.05). ^⸸^ Significantly different from Day 0 (*p* ≤ 0.05). Note. Day 0 is prior to participant’s supplementation, 0 min is after 3 days of supplementation, and 60 min, 120 min, and 180 min are after the 4th acute dose (4 consecutive days of supplementation) of the investigational product.

**Table 3:** Feelings of affect via VAS at baseline (Day 0) and testing visits (Day 4).

		Time				
	Group	Day 0	0 Min (Day 4)	60 Min (Day 4)	120 Min (Day 4)	180 Min (Day 4)
Energy (cm)	PLA	5.6 ± 1.9	5.6 ± 1.9	5.8 ± 2.0	6.1 ± 1.8	6.1 ± 1.9 *
	ML	5.5 ± 2.0	5.8 ± 2.1	6.2 ± 1.9	6.2 ± 2.2	6.5 ± 1.9 *
Sustained energy (cm)	PLA	5.7 ± 1.8	5.6 ± 1.9	5.8 ± 2.0	6.2 ± 1.7 *	6.1 ± 1.9 *
	ML	5.6 ± 2.0	5.7 ± 2.1	6.2 ± 1.9 *	6.3 ± 2.2	6.5 ± 2.0 *
Mental Stamina (cm)	PLA	5.8 ± 1.8	5.6 ± 2.0	6.0 ± 1.9	6.1 ± 1.7 *	6.0 ± 1.9 *
	ML	5.7 ± 1.9	6.0 ± 1.9	6.3 ± 1.9	6.3 ± 2.1	6.6 ± 1.9
Focus (cm)	PLA	5.9 ± 1.9	6.0 ± 1.9	6.0 ± 2.0	6.2 ± 1.8	6.2 ± 1.9
	ML	5.9 ± 1.8	6.0 ± 2.0	6.4 ± 1.8	6.5 ± 2.0	6.7 ± 2.0
Drive (cm)	PLA	5.7 ± 1.9	5.7 ± 2.0	6.0 ± 1.9	6.3 ± 1.7 *	6.0 ± 1.8
	ML	5.8 ± 1.9	5.9 ± 1.9	6.1 ± 2.1	6.2 ± 2.0	6.4 ± 2.1
Vigor (cm)	PLA	5.8 ± 1.8	5.7 ± 2.0	6.1 ± 2.0	6.3 ± 1.8 *	5.9 ± 1.8
	ML	5.8 ± 2.0	5.8 ± 2.0	6.2 ± 2.0	6.3 ± 1.9	6.4 ± 2.0 *
Positive Outlook (cm)	PLA	6.8 ± 1.7	6.4 ± 1.9 ^⸸^	6.7 ± 1.8	7.0 ± 1.7 *	6.8 ± 1.9
	ML	6.6 ± 1.9	6.4 ± 2.0	6.8 ± 1.9	7.0 ± 1.7	7.0 ± 1.7 *
Well-being (cm)	PLA	7.0 ± 1.7	6.6 ± 1.8	6.9 ± 1.8	7.0 ± 1.6	6.9 ± 1.8
	ML	6.8 ± 2.0	6.4 ± 1.7	6.8 ± 1.7	7.3 ± 1.6 *	7.2 ± 1.6 *
Ability to Tolerate Stress (cm)	PLA	6.1 ± 1.9	5.8 ± 2.1	6.0 ± 2.0	6.1 ± 2.0	5.8 ± 2.0
	ML	6.1 ± 2.0	5.7 ± 2.0	6.2 ± 2.1	6.4 ± 1.9 *	6.4 ± 1.9

PLA: Placebo. ML: Methylliberine. * Statistically significant difference from 0 min time point (*p* ≤ 0.05). ^⸸^ Significantly different from Day 0 (*p* ≤ 0.05). Note. Day 0 is prior to participant’s supplementation, 0 min is after 3 days of supplementation, and 60 min, 120 min, and 180 min are after the 4th acute dose (4 consecutive days of supplementation) of the investigational product.

**Table 4:** Vitals at baseline (Day 0) and testing visits (Day 4).

	Heart Rate (bpm)		SBP (mmHg)		DBP (mmHg)	
Time	PLA	ML	PLA	ML	PLA	ML
Day 0	71.8 ± 13.7	71.4 ± 11.9	122.0 ± 12.2	118.6 ± 12.9	77.1 ± 7.1	76.6 ± 10.2
0 min (Day 4)	71.6 ± 11.4	69.8 ± 11.2	120.6 ± 12.8	121.4 ± 16.0	75.3 ± 9.3	77.3 ± 10.0
60 min (Day 4)	66.5 ± 13.5	66.4 ± 11.8	118.2 ± 12.5	117.9 ± 12.9	76.2 ± 9.2	76.9 ± 8.8
120 min (Day 4)	66.4 ± 13.5	64.7 ± 9.9	119.2 ± 11.9	120.6 ± 13.5	79.5 ± 11.7 *	75.6 ± 9.1
180 min (Day 4)	65.7 ± 12.0	66.0 ± 10.9	121.6 ± 11.7	120.0 ± 12.5	77.8 ± 9.8	77.2 ± 9.1

PLA: Placebo. ML: Methylliberine. SBP: Systolic blood pressure; DBP: Diastolic blood pressure; and PL: Placebo. * Significantly different from 0 min (*p* ≤ 0.05). Note. Day 0 is prior to participant’s supplementation, 0 min is after 3 days of supplementation, and 60 min, 120 min, and 180 min are after the 4th acute dose (4 consecutive days of supplementation) of the investigational product.

## Data Availability

Restrictions apply to the availability of these data. The data that support the findings of this study are available by reasonable request from the authors upon permission from the sponsor (Compound Solutions).
